# Supplementary material for: Identification of Mobile Colistin Resistance Gene mcr-10 in Disinfectant and Antibiotic Resistant Escherichia coli from Disinfected Tableware
Source: Antibiotics (Basel). 2022 Jul 1;11(7):883. doi: 10.3390/antibiotics11070883 (PMC9311939; doi:10.3390/antibiotics11070883)
Supplement: Supplementary file 1 [file antibiotics-11-00883-s001.zip › Supplementary Files.pdf]

Table S1. Information of plasmids with the five highest matched plasmids to pMCR_10_2641 in NCBI.

Subject ID	Length (bp)	Query cover (%)	Identity (%)	Dscription
CP040383	194556	45.34	99.99	*Escherichia coli* strain A1_180 plasmid unnamed2
CP025740	160682	43.43	100	*Escherichia coli* strain Ec40 plasmid unnamed
CP041047	301036	32.22	98.34	*Citrobacter sp.* CF971 plasmid pBM527-1,
CP026206	191344	40.58	100	*Escherichia coli* strain ECONIH5 plasmid pECO-cbb3,
CP023978	213341	36.69	96.43	*Klebsiella variicola* strain X39 plasmid pX39-1,

Table S2. Plasmids carrying *mcr-10* gene on NCBI.

Species	Strain	Plasmid Name	Plasmid Replicons	Size (bp)	Country	Year	Accession no.
Enterobacter roggenkampii	WCHER090065	pMCR10_090065	IncFIA	71775	China	2019	CP045065
Cronobacter sakazakii	145005	pMCR10_145005	IncFIB	120500	China	2021	JABTXY010000030
Enterobacter roggenkampii	Ecl_20_981	pECL981-1	IncFIB	161986	China	2020	CP048651
Enterobacter roggenkampii	Er_20_983	pEr983-1	IncFIB	100102	China	2020	CP060738
Raoultella ornithinolytica	FDAARGOS_431	unnamed1	IncFIB	231294	USA	2017	CP023893
Citrobacter freundii	B38	pOZ172	IncFIB	127005	China	2001	CP016763
Raoultella ornithinolytica	NUITM-VR1	pNUITM-VR1_2	IncFIB	261835	Japan	2021	AP025011
Enterobacter hormaechei	ECC59	pECC59-2	ND	64293	China	2021	CP080472

Enterobacter cloacae	En37	pEN37S	IncFIB	70277	Japan	2021	AP024497
Enterobacter roggenkampii	STW0522-66	pSTW0522-66-1	IncFIB	324199	Japan	2020	AP022466
Enterobacter kobei	STW0522-51	pSTW0522-51-1	ND	159829	Japan	2020	AP022432
Enterobacter asburiae	RHBSTW-01009	pRHBSTW-01009_2	IncFIB	70650	UK	2020	CP056127
Enterobacter cloacae	RHBSTW-00399	pRHBSTW-00399_2	IncFII	137623	UK	2020	CP056561
Enterobacter roggenkampii	OIPH-N260	pN260-2	IncFIB	244996	Japan	2020	AP023449
Enterobacter roggenkampii	YK16	pYK16-mcr-10	IncFII	117855	China	2020	MT468575
Enterobacter sp.	18A13	pECC18A13-1	lncFII	150509	Japan	2019	AP019635
Enterobacter sp.	RHBSTW-00175	pRHBSTW-00175_3	IncFIB	68715	UK	2020	CP055932
Enterobacter cloacae	PIMB10EC27	pEC27-2	IncFIA	84602	Viet Nam	2018	CP020091
Klebsiella quasipneumoniae	SB610	pKqs_SB610_4	IncFIB	124980	Australia	2021	CP084774
Klebsiella pneumoniae	INF133-sc-2279960	pINF133-sc-2279960_5	IncFII	120029	Australia	2020	LR890193

ND, not detected; bp, base pair.

Table S4. The PT-qPCR oligonucleotide primer sequences used in this study.

Primer use and target gene	Nucleotide sequence (5′→3′)	Amplicon size (bp)	Annealing temperature (℃)
16s	F: TCCACGATTACTAGCGATTC<br>R: AGCAAGCGGACCTCATAA	87	55
mcr-10	F: ACCTGCTATGACGATGTTAT<br>R: TGAAGTGACGATGCTCTG	124	55

F, forward; R, reverse; bp, base pair

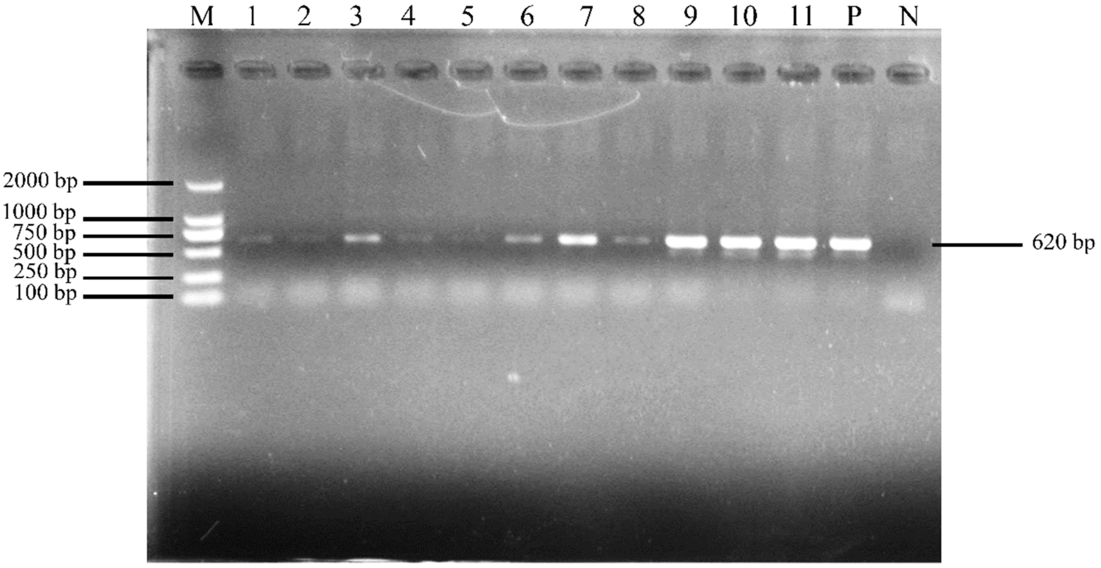

Figure S1. Agarose gel electrophoresis of the *mcr-10* PCR product from DNA extracts of the transconjugants. M lane is marker; lanes 1-11 are transconjugants; P lane is *mcr-10* carrying strain EC2641 as a positive control; N lane is using recipient bacterium J53 as a negative control. *mcr-10* amplification product size is 620 bp.

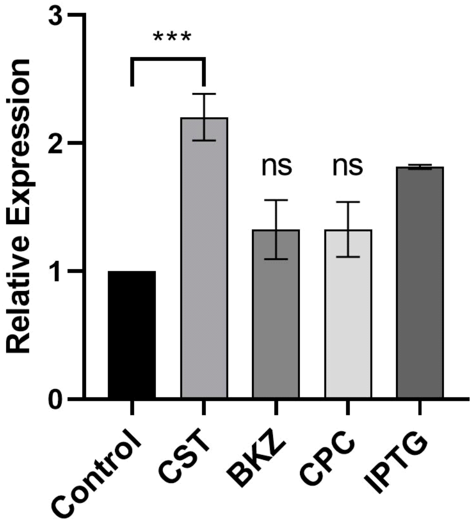

Figure S2. Relative expression of the resistance gene *mcr-10* in *Escherichia coli* EC2641 (the original host of *mcr-10* gene) under colistin, benzalkonium chloride, Cetylpyridinium Chloride and IPTG stress. CST, colistin; BKZ, benzalkonium chloride; CPC, Cetylpyridinium Chloride; IPTG, Isopropyl-β-D-thiogalactopyranoside. and antibiotic free LB was used as control. The data are shown as the means ± SD of the results from three individual assays. ns, $p > 0.05$; ***, $p < 0.001$.
